# Letter to the Editor Regarding the Article: “Enterovirus and Paraechovirus Meningitis in Neonates: Which Is the Difference?”

**DOI:** 10.1177/00099228241265915

**Published:** 2024-07-24

**Authors:** Giacomo Brisca, Tommaso Bellini, Marcello Mariani

**Affiliations:** 1 Neonatal and Pediatric Intensive Care Unit, and Intermediate Care Unit, Emergency Department, IRCCS Istituto Giannina Gaslini, Genoa, Italy; 2 Emergency Room and Pediatric Emergency Medicine Unit, Emergency Department, IRCCS Istituto Giannina Gaslini, Genoa, Italy; 3 Pediatric Infectious Diseases Unit, Department of Pediatrics, IRCCS Istituto Giannina Gaslini, Genoa, Italy

**Keywords:** parechovirus, enterovirus, newborns, central nervous system, viral infection

**Dear Editor**

We really appreciate the recent manuscript authored by Picone and colleagues,^1^ as it addresses a topic of great interest to us.

The authors describe a series of 18 newborns with meningitis by enterovirus (EV) and parechovirus (HpEV) with a favorable outcome.

However, we believe that 2 main points deserve discussion.

First, no patients in their series underwent magnetic resonance imaging (MRI) of the brain. In our opinion, this represents a strong limitation, as brain abnormalities are commonly reported in infants with HpEV and EV infections (36% in our experience,^2^ similar to other studies).^3^

Furthermore, as the authors state, recent evidence supports a correlation between the severity of neurological outcomes in HpEV central nervous system (CNS) infections and white matter changes observed by MRI.^4^

Therefore, we suggest that all infants with EV or HpEV CNS infection be considered for an MRI, especially if a brain ultrasonography is inconclusive or equivocal or if there are symptoms suggestive of CNS involvement.

The second point concerns the possible distinguishing features between EV and HpEV infection. We agree with the authors that distinguishing between the 2 etiologies is often not clinically possible and that some subtle laboratory differences can be emphasized, such as the mild increase in C-reactive protein detected in patients with EV infection. Unfortunately, Picone et al did not report on ferritin levels in their patients. As reported also by others,^5^ in our study,^2^ we found a significantly increased ferritin level in neonates with HpEV infection. In our opinion, hyper-ferritinemia may represent a specific diagnostic clue for HpEV infection and should be included in the diagnostic approach to febrile infants. In addition, ferritin monitoring may allow early interception of the most fearful complication of HpEV infection, namely hemophagocytic lymphohistiocytosis, and initiation of appropriate immunomodulatory treatment. Further research will help us confirm our observations.

## Author Contributions

**GB:** Writing – review & editing, Visualization, Validation, Supervision, Methodology, Investigation, Formal analysis, Data curation, Conceptualization.

**TB:** Writing – original draft, Investigation, Formal analysis, Data curation, Conceptualization.

**MM:** Writing – review & editing, Investigation, Formal analysis, Data curation, Methodology, Conceptualization.
